# First record of the genus *Pectapalochrus* Tshernyshev, 2016 (Coleoptera, Malachiidae) from China

**DOI:** 10.3897/BDJ.11.e104877

**Published:** 2023-05-10

**Authors:** Junbo Tong, Sergei E. Tshernyshev, Haoyu Liu, Yuxia Yang

**Affiliations:** 1 The Key Laboratory of Zoological Systematics and Application, School of Life Science, Institute of Life Science and Green Development, Hebei University, 071002, Baoding, China The Key Laboratory of Zoological Systematics and Application, School of Life Science, Institute of Life Science and Green Development, Hebei University, 071002 Baoding China; 2 Institute of Systematics and Ecology of Animals, Siberian Branch of the Russian Academy of Sciences, Frunze Street 11, 630091, Novosibirsk, Russia Institute of Systematics and Ecology of Animals, Siberian Branch of the Russian Academy of Sciences, Frunze Street 11, 630091 Novosibirsk Russia; 3 Tomsk State University, Lenina prospekt 36, 634050, Tomsk, Russia Tomsk State University, Lenina prospekt 36, 634050 Tomsk Russia

**Keywords:** new record, Apalochrini, taxonomy, China, Cleroidea

## Abstract

**Background:**

*Pectapalochrus* Tshernyshev, 2016 is a small genus belonging to the tribe Apalochrini in the family Malachiidae (Coleoptera, Cleroidea), with four currently known species: *P.omega* (Evers, 1965) from Mongolia, *P.armenus* (Iablokoff-Khnzorian, 1978) from Armenia, *P.pictus* (Motschulsky, 1860) from Russia and *P.pectinicornis* (Erichson, 1840) from Russia and Mongolia.

**New information:**

*Pectapalochrus* Tshernyshev, 2016 is newly recorded from China upon the discovery of *P.omega* (Evers, 1965) from Ningxia Autonomous Region. The morphological characters of the adult are re-described in detail with illustrations of external appearance and relevant characters.

## Introduction

The genus *Pectapalochrus* was proposed by Tshernyshev (2016a) on the basis of four species separated from *Dromanthomorphus* Pic, 1921 of Malachiidae (Majer 2002, Mayor 2007, Bocakova et al. 2012). *Pectapalochrus* was attributed to Apalochrini due to its long and pectinate antenna in male with the small pedicel almost hidden by the scape and can be distinguished from *Dromanthomorphus* by its slender fore and middle legs and simple metaventrite and pygidium (apical tergite) without appendages or apophyses (Tshernyshev 2016a). In contrast, *Dromanthomorphus* has swollen fore and middle legs and modified metaventrite possessing apophysis directed forwards (Tshernyshev 2016b). Besides, the members of *Pectapalochrus* are all constricted to the Palaearctic Region (Tshernyshev 2016a), while *Dromanthomorphus* is in the Oriental Region (Tshernyshev 2016b).

To date, the genus *Pectapalochrus* is comprised of four species distributed in Armenia, Mongolia and Russia (Tshernyshev 2016a). In the present study, some specimens of this genus were discovered from China and identified as *P.omega* (Evers, 1965), which represents the first record of *Pectapalochrus* from the Chinese fauna. *Pectapalochrusomega* is the type species of the genus *Pectapalochrus* and shows an obvious elytral pattern variation, which is very rare in the family Malachiidae (Evers 1969, Tshernyshev 2016a). Hence, a more detailed description of the species, as well as illustrations of external appearance and special characters with a distribution map are provided. The female ovipositor, pygidium (apical tergite) and ultimate abdominal ventrite (apical sternite) of *P.omega* are illustrated and described for the first time.

## Materials and methods

In this study, Malachiidae beetles are considered as a family (Majer 1994, Majer 2002, Mayor 2007, Bocakova et al. 2012, Constantin 2021). A new system, based on a cladistic study of several species from different Cleroidea families, resulted in the Malachiidae being assigned to a subfamily of the Melyridae sensu lato (Gimmel et al. 2019). To build a stable system, further phylogenetic analysis with more representatives of this family including the determination of typical molecular characteristics is required.

For descriptions, special male structures and genitalia were studied. The term “special male structures” is not analogous to the term “Excitatoren”, that means different kinds of structures located in different parts of the male body of soft-winged flower beetles and bearing ducts of pheromone glands necessary for female attraction and successful copulation (Evers 1956, Evers 1963, Evers 1988, Matthes 1962). The “special male structures” includes all typical parts of the male, irrespective of their having pheromone glands or not.

Terminology of terminalia morphology is according to Lawrence et al. (2010), namely (in comparison with previously used terms): pygidium for apical tergite, ultimate abdominal ventrite for apical sternite and endophallus for the inner sac of the aedeagus.

The specimens examined in this study are deposited in Museum of Hebei University, Baoding, China (MHBU). The specimen had its abdomen detached and soaked in 10% solution of sodium hydroxide (NaOH) by boiling for several minutes. Ovipositor was dyed with haematoxylin. Genitalia were dissected, cleaned and transferred to glycerol on slides and photographed with a LEICA DFC450 colour digital camera attached to the LEICA M205 A microscope. LAS V.4.7 software was used to capture genitalia images. External morphology was observed with the Nikon SMZ1500 stereomicroscope. Images of adults were taken with a Canon EOS 80D digital camera and stacked in Helicon Focus 7. The final plates were prepared in Adobe Photoshop CS 6.0.

## Taxon treatments

### Pectapalochrus

Tshernyshev, 2016

33159C26-C909-5FC4-8580-AC92AB0E3F47

Pectapalochrus Tshernyshev, 2016 - Tshernyshev 2016a: 349.
Pectapalochrus Apalochrus omega Evers, 1965

#### Diagnosis

Body medium-sized, ranging from 2.8 to 4.7 mm in length. Antennae flabellate or strongly serrate, antennomere 2 very small and not conspicuous, antennomere 3 triangular (Fig. 2b). Tarsomere 2 of the fore tarsi bearing a special comb in male (Fig. 2g). Ultimate abdominal ventrite (apical sternite) shortened and divided in both male and female (Fig. 2h, Fig. 3b).

#### Distribution

China (new faunistic record: Ningxia), Russia, Mongolia, Armenia.

### Pectapalochrus omega

(Evers, 1965)

895E6F47-4005-58D0-95A6-61BAAE7A572D

*Apalochrusomega* Evers, 1965 - Evers 1965: 149 (type locality: Mongolia).
*Flabellapalochrusomega* (Evers, 1965): Evers, 1987 - Evers 1987: 59.
*Dromanthomorphusomega* (Evers, 1965): Wittmer, 1990 - Wittmer 1990: 112; Mayor, 2007 - Mayor 2007: 416.
*Pectapalochrusomega* (Evers, 1965): Tshernyshev, 2016 - Tshernyshev 2016a: 351.
*Apalochrusboops* Evers, 1968 - Evers 1968: 33. Synonymized by Evers 1969: 188.

#### Materials

- **Type status:** Other material. **Occurrence:** recordedBy: Guodong Ren; individualCount: 3; sex: 1 male, 2 females; lifeStage: adult; occurrenceID: FC88DCF7-9E42-5A67-8D3C-E8A8250B640F; **Location:** country: China; stateProvince: Ningxia; county: Zhongwei; municipality: Shapotou; **Event:** year: 1987; month: 6; day: 8; **Record Level:** institutionID: Museum of Hebei University; institutionCode: MHBU
- **Type status:** Other material. **Occurrence:** recordedBy: Guodong Ren; individualCount: 1; sex: 1 male; lifeStage: adult; occurrenceID: ECC90ED4-65B2-5B5E-8A71-FA2142F862B8; **Location:** country: China; stateProvince: Ningxia; county: Zhongwei; locality: Gantang, Shapotou; **Event:** year: 1987; month: 6; **Record Level:** institutionID: Museum of Hebei University; institutionCode: MHBU
- **Type status:** Other material. **Occurrence:** recordedBy: Kang Lou; individualCount: 2; sex: 2 female; lifeStage: adult; occurrenceID: F444DB70-F6B2-5201-ACEA-6C3381451A88; **Location:** country: China; stateProvince: Ningxia; county: Yanchi; locality: Qingyangjing; verbatimElevation: 1469; verbatimLatitude: 37.96°N; verbatimLongitude: 107.18°E; **Event:** year: 2017; month: 5; day: 19; **Record Level:** institutionID: Museum of Hebei University; institutionCode: MHBU
- **Type status:** Other material. **Occurrence:** recordedBy: Kang Lou; individualCount: 1; sex: 1 female; lifeStage: adult; occurrenceID: 5CE8568D-3B16-5F49-8558-C1810667F443; **Location:** country: China; stateProvince: Ningxia; county: Yanchi; locality: Xijingtan; verbatimElevation: 1343; verbatimLatitude: 37.87°N; verbatimLongitude: 107.57°E; **Event:** year: 2017; month: 5; day: 27; **Record Level:** institutionID: Museum of Hebei University; institutionCode: MHBU
- **Type status:** Other material. **Occurrence:** recordedBy: Kang Lou; individualCount: 1; sex: 1 male; lifeStage: adult; occurrenceID: 2625194C-EB6B-5C7A-BE4F-B1021003ED0C; **Location:** country: China; stateProvince: Ningxia; county: Yanchi; locality: Zhouzhuangzi; verbatimElevation: 1452; verbatimLatitude: 37.74°N; verbatimLongitude: 107.36°E; **Event:** year: 2017; month: 6; day: 10; **Record Level:** institutionID: Museum of Hebei University; institutionCode: MHBU

#### Description

**Male.** Length of body 3.5–4.7 mm, width at widest part of elytra 1.5–2.3 mm and at the base of elytra 1.2–1.6 mm.

**Figure 1.**
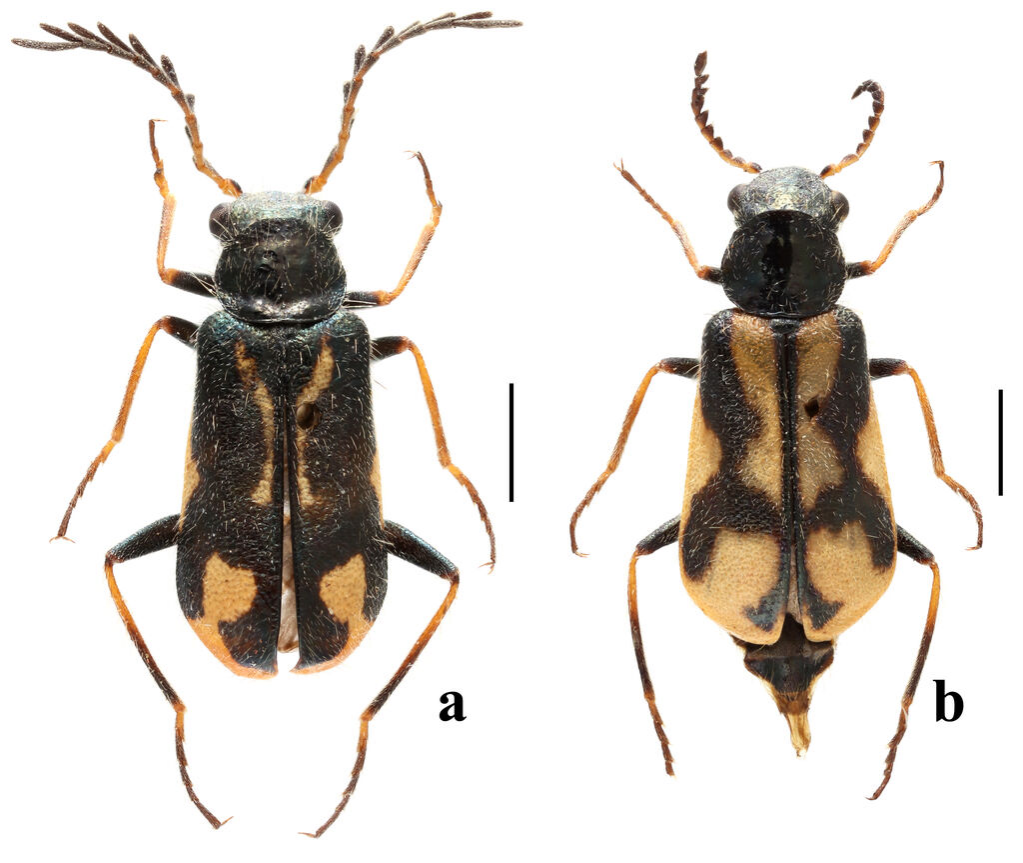
*Pectapalochrusomega* (Evers, 1965), habitus: **a** male, dorsal view; **b** female, dorsal view. Scale bars: 1.0 mm.

Head capsule black. Antennae yellow to black: antennomere 1 yellow with a dark spot on inner side; 2 entirely yellow; 3–8 yellow to black; 9–11 entirely black. Mouthparts black. Pronotum black. Elytron with variable black and yellow markings, from black with a small yellow spot near apex to yellow with two black stripes at humerus and apical third. Scutellar shield black. Legs yellow to black (Fig. 1a). Ventral surface black. Vesicles yellow. Dorsum with double pubescence consisting of adpressed pubescence and sparse white stiff bristles. Sculptures evenly punctuated, stronger on elytra than on other parts.

**Figure 2.**
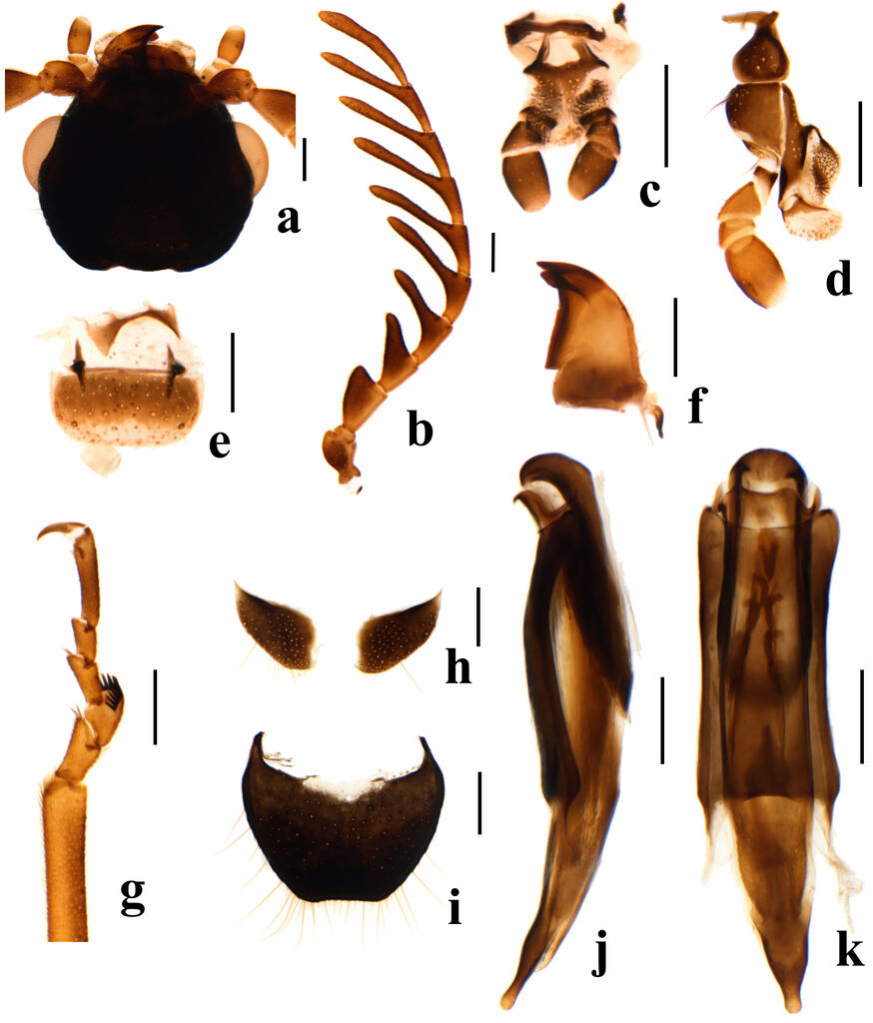
Main characters of *Pectapalochrusomega* (Evers, 1965), male. **a** head; **b** antenna; **c** labium; **d** maxilla; **e** labrum; **f** mandible; **g** fore tarsi; **h** ultimate abdominal ventrite (apical sternite); **i** pygidium (apical tergite); **j** male genitalia, lateral view, **k** male genitalia, ventral view. Scale bars: 0.2 mm.

Head almost as wide as pronotum (Fig. 1a). Frons flat, interocular area not depressed and lacking protuberances (Fig. 2a). Antennae long and flabellate, projecting beyond middle of elytra; antennomere 1 widened and club-shaped; 2 small and almost completely hidden by 1; 3 enlarged, right triangular; 4–10 serrate to flabellate and almost equal in length; 11 elongate and slender (Fig. 2b). Labial palps with 3 palpomeres, apical palpomere subcylindrical (Fig. 2c). Maxilla with cardo short; palpomere 2 triangular; palpomere 3 small; palpomere 4 cylindrical and robust (Fig. 2d). Clypeus distinct and membranous (Fig. 2e). Mandible robust, with a subapical tooth just behind apical tooth (Fig. 2f).

Pronotum subrounded, lateral sides evenly rounded, anterior margin slightly convex, posterior margin straight, with a distinct transverse depression at base (Fig. 1a).

Scutellar shield small and transverse, with smoothed edges (Fig. 1a).

Elytra subparallel, widened behind the base and evenly rounded at apices, base of elytra distinctly wider than pronotum. Humeri distinct, slightly protruding. Elytral apices evenly rounded (Fig. 1a).

Hind wings normally developed.

Legs slender. Hind femora not reaching elytral apices. All tibiae thin and straight (Fig. 1a). All tarsi with 5 tarsomeres; tarsomere 2 of the anterior tarsi with comb extending over the segment; tarsomere 5 longest and tarsomere 4 shortest in all legs. Claws long and sharp, with small rounded bases (Fig. 2g).

Metathorax simple, lacking appendages. Pygidium transverse, with almost straight distal side (Fig. 2i). Ultimate abdominal ventrite bilobed, short and transverse (Fig. 2h). Tegmen elongate; aedeagus wide, approximately parallel-sided for basal 2/3 in ventral view, then slightly narrowing to blunt apex; endophallus with two rows of small spines (Fig. 2j–k).

**Female.** Length of body 3.5–4.6 mm, width at widest part of elytra 1.6–2.2 mm and at the base of elytra 1.1–1.6 mm.

Similar to male species, except for antennae short and serrate, fore-tarsi lacking comb, pronotum with an inconspicuous transverse depression at base (Fig. 1b).

**Figure 3.**
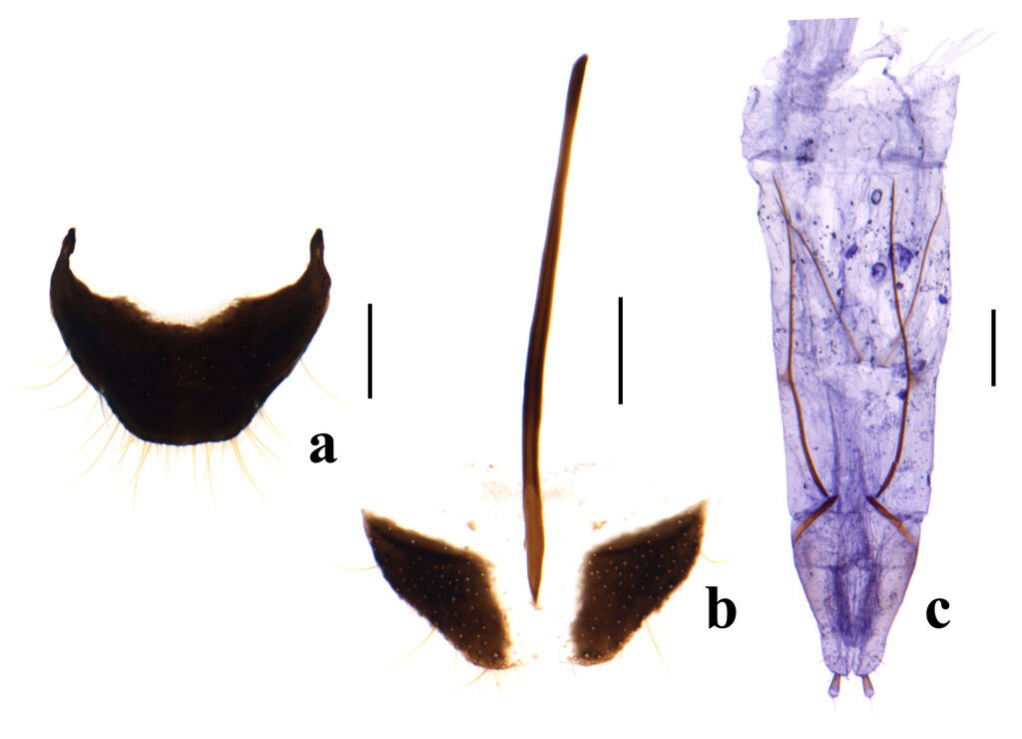
*Pectapalochrusomega* (Evers, 1965), female: **a** pygidium (apical tergite); **b** ultimate abdominal ventrite (apical sternite); **c** ovipositor. Scale bars: 0.2 mm.

Pygidium sub-trapezoid with apical margin almost straight (Fig. 3a). Ultimate abdominal ventrite divided, with long spiculum ventrale (Fig. 3b). Ovipositor elongate and membranous (Fig. 3c).

#### Diagnosis

This species is similar to *P.pectinicornis* (Erichson, 1840) in entirely black pronotum and yellow to black antennae and legs, but can be distinguished from the latter by the elytra with variable black and yellow markings, antennomere 3 scalene triangular in male and subcylindrical in female (Fig. 1, Fig. 2b). In *P.pectinicornis*, elytra are black with wide yellow rounded spots at apices, antennomere 3 is equicrural triangular in male and triangular in female (Tshernyshev 2016a).

#### Distribution

**Figure 4.**
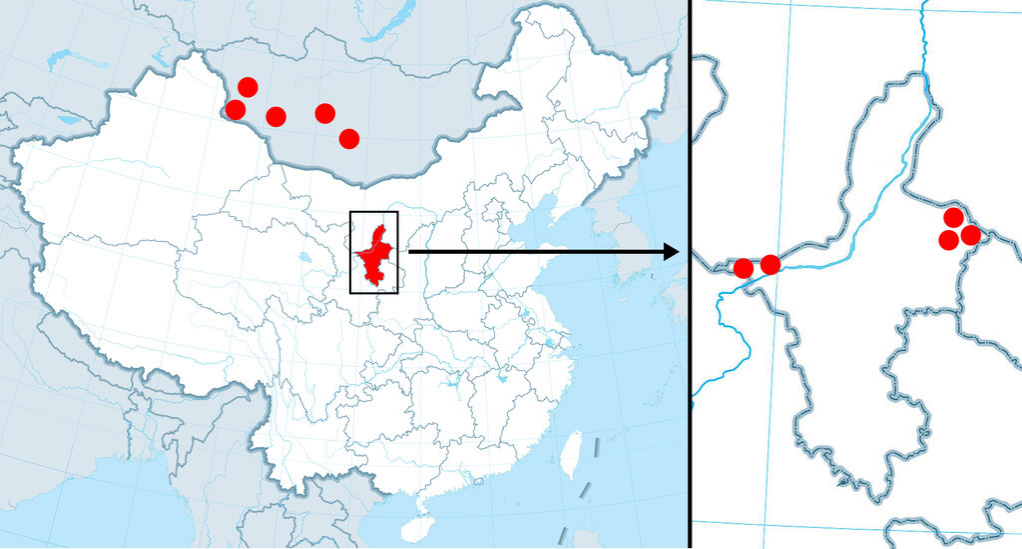
Distribution map of *Pectapalochrusomega* (Evers, 1965) in Mongolia and Ningxia Autonomous Region of China (red circle).

China (Ningxia); Mongolia (Fig. 4).

## Supplementary Material

XML Treatment for Pectapalochrus

XML Treatment for Pectapalochrus omega
